# Evaluation of clinically applied treatment beams with respect to bunker shielding parameters for a Cyberknife M6

**DOI:** 10.1002/acm2.12215

**Published:** 2017-11-10

**Authors:** Dominik Henzen, Daniel Schmidhalter, Claudia Christina Zanella, Werner Volken, Paul‐Henry Mackeprang, Marco Malthaner, Michael Karl Fix, Peter Manser

**Affiliations:** ^1^ Division of Medical Radiation Physics Department of Radiation Oncology, Inselspital Bern University Hospital University of Bern Bern Switzerland; ^2^ Institute for Biomedical Engineering ETH Zürich Zürich Switzerland

**Keywords:** Cyberknife, radiation protection, SBRT

## Abstract

Compared to a conventional linear accelerator, the Cyberknife (CK) is a unique system with respect to radiation protection shielding and the variety and number of non‐coplanar beams are two key components regarding this aspect. In this work, a framework to assess the direction distribution and modulation factor (MF) of clinically applied treatment beams of a CyberKnife M6 is developed. Database filtering options allow studying the influence of different parameters such as collimator types, treatment sites or different bunker sizes. A distribution of monitor units (MU) is generated by projecting treatment beams onto the walls, floor and ceiling of the CyberKnife bunker. This distribution is found to be highly heterogeneous and depending, among other parameters, on the bunker size. For our bunker design, 10%–13% of the MUs are delivered to the right and left wall, each. The floor receives more than 64% of the applied MUs, while the wall behind the patient's head is not hit by primary treatment beams. Between 0% and 5% of the total MUs are delivered to the wall at the patient's feet. This number highly depends on the treatment site, e.g., for extracranial patients no beams hit that wall. Collimator choice was found to have minor influence on the distribution of MUs. On the other hand, the MF depends on the collimator type as well as on the treatment site. The MFs (delivered MU/prescribed dose) for all treatments, all MLC treatments, cranial and extracranial treatments are 8.3, 6.4, 7.7, and 9.9 MU/cGy, respectively. The developed framework allows assessing and monitoring important parameters regarding radiation protection of a CK‐M6 using the actually applied treatment beams. Furthermore, it enables evaluating different clinical and constructional situations using the filtering options.

## 1 INTRODUCTION

In clinical practice of radiation oncology, staff members as well as persons of the general public need to be protected from ionizing radiation and dose limits according to locally relevant legal laws have to be fulfilled. This leads to the typical task of a medical physicist to optimize the design of bunkers such that radiation protection issues are managed, while keeping the corresponding costs and resources of the bunker construction as low as possible. This is a challenging task for dedicated delivery systems such as the Cyberknife (CK) system (Accuray Inc, Sunnyvale CA, USA). In general, the dose given to a person can be expressed as follows:

(1) $$D_{pers}=\sum_{i}{\dot{D}}_{i}*t_{i}$$

where: $\sum_{i}$ = sum over all exposures i

${\dot{D}}_{i}$ = dose rate due to exposure i

$t_{i}$ = time duration of exposure i

Based on eq. (1) one approach to realize practical radiation protection is the reduction of $t_{i}$. Another possibility is to place attenuating material between the source of radiation and the person, e.g., build a bunker, which reduces the dose rate. This bunker shielding problem can be separated into primary and secondary barriers.^1^ For the CK system, the primary beam can point in almost any direction such that almost everywhere a primary barrier is needed for radiation protection purposes.^2^ Secondary beams are related to leakage radiation as well as to scattered radiation and with respect to this, the CK is not very much different from standard delivery systems such as linear accelerators (linacs).

It is important for the motivation of this work that, a few years ago, a new CK model (so‐called M6 model) has been released, which differs from the previous versions^3^ in geometrical and dose delivery aspects. First, the CK‐M6 version encompasses a more symmetric arrangement between the delivery robot and the couch such that the beam arrangements are also more symmetric than for previous versions. Moreover, the CK‐M6 is equipped with a multileaf collimator (MLC) increasing the flexibility and versatility.^4^ It is thus the aim of this work to investigate whether shielding considerations for both, primary and secondary radiation have to be revised when switching from a conventional linac to the CK‐M6. Furthermore, this work assesses the impact of the novel MLC on the required radiation shielding of the CK by analyzing clinically applied treatment beams.

## 2 MATERIALS AND METHODS

### 2.A Evaluated situation

For this study, clinical treatment plans delivered by a CK‐M6 at our center were analyzed. This robot‐based stereotactic radiation therapy system was initially equipped with a fixed field size interchangeable cone collimators (Fix) and an Iris (Iris) collimator system^5^ allowing the collimation of the beam shaped into circles or dodecagons, respectively. The twelve available diameters or circumdiameters for these collimators range from 5 to 60 mm, defined at a source‐to‐axis distance (SAD) of 800 mm. Since 2015, a third collimation device, the MLC is available in our clinic allowing to deliver field sizes up to 100 × 115 mm (again defined at SAD of 800 mm). For the CK‐M6, a treatment plan consists of several beams, which originate from a discrete set of robot positions, called nodes, and point toward different positions within the target volume.

For this study, we retrospectively analyzed a database of 364 CK treatments performed at our center. Altogether, these patients received 1115 treatment fractions, which lead to a total number of 166'125 applied beams. An overview of treated tumor sites and used collimator types is provided in Table 1.

**Table 1:** Treatment site and used collimator for the included patients

	Cranial	Extracranial					All
	Head	Lung	Liver	Prostate	Spine	Other	All
Fix	156	0	0	1	6	0	163
Iris	99	18	15	14	10	24	180
MLC	6	1	7	6	0	1	21
All	261	19	22	21	16	25	364

Allocation of the included patients to the respective treatment site and used collimator system.

**Figure 1:**
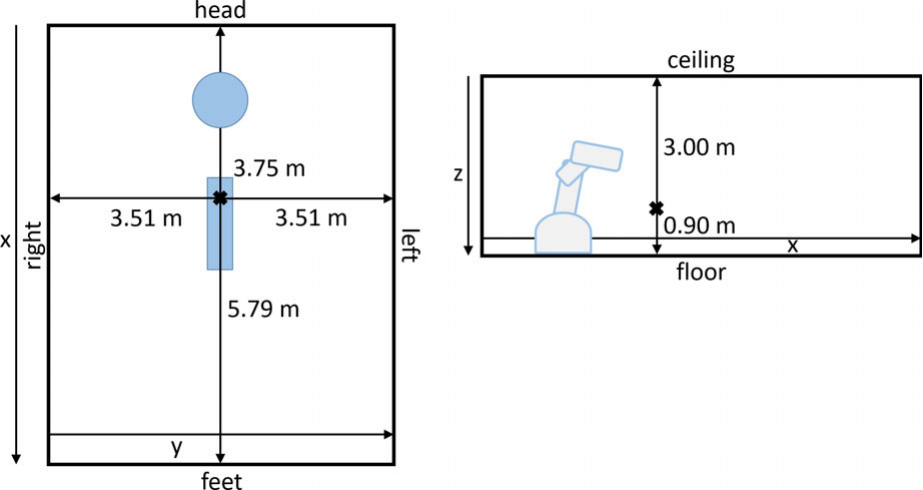
CK bunker room coordinate system. Sketch of the CK bunker room with indicated dimensions and coordinate system. The cross represents the origin of the coordinate system. The blue circle and the CK scheme indicate the position of the robot inside the room. The blue rectangle indicates the treatment couch. The labels for the walls and the ceiling/floor will be referenced to throughout this work.

The primarily used bunker design in this work is illustrated by Fig. 1. It resembles the footprint of our bunker and is referred by its footprint size of 9.5 × 7.0 m^2^. For reasons of simplicity, the entrance barrier was neglected in this study and dimensions in left and right directions were adapted to be symmetrical. Figure 1 also shows the position of the robot inside the bunker and the notations for the different walls, the floor and the ceiling used throughout this work.

### 2.B Framework

The CK data management system stores information about all applied beams into a database. This information can be extracted as a log file in xml format. In a first step, the newly developed framework reads for each patient the prescribed dose and the log files from all delivered patient treatments and creates a file containing the following information for each delivered beam: robot position as well as beam direction, number of delivered monitor units (MUs), collimator system used, applied tracking mode, and field size at SAD 800 mm. Note, that for the MLC the field size is defined as the area that is not covered by the leaves. All those parameters, together with the treatment site, which is mapped from a separate database, are recorded and stored into the treatment list. This treatment list is then purged from all sensitive information and serves as a completely anonymized repository of the clinically applied treatments. In a second step, the CK bunker is defined as a rectangular geometry with freely selectable dimensions.

In order to extract and combine the available information, several routines are implemented in Python (Python Software Foundation, 149 Hampton, NH, www.python.org), version 3.5. For all routines, data may be filtered according to collimator type or treatment site. These routines allow an analysis of the mentioned parameters as well as projecting the beams onto the inner surfaces of the considered bunker design by simply ray‐tracing the beam on its central beam axis.

### 2.C Evaluated parameters

All beams included in the treatment list are weighted with the corresponding MUs, projected onto the inner surface of the bunker (represented by 50 × 50 cm pixels) and the distribution of the applied MUs are evaluated. This includes the MU distribution for each single barrier as well as a top view of the bunker, resulting from integrating all the applied MUs of the walls along the z‐axis (as defined in Fig. 1). For further evaluations, the treatment list is filtered in order to investigate the influence of the different treatment sites and collimator systems on previously described MU distributions for the bunker barrier (cf. Table 1). To evaluate the influence of room size on the MU distribution for different bunker geometries, different bunker sizes are considered. In addition to the minimal allowed bunker floor size of approximately 6.4 × 4.8 m^2^ and the recommended size of 7.3 × 6.4 m^2^, also a rather small although flexible bunker, and large squared sized bunker with footprints of 6.4 × 6.4 m^2^ and 10.0 × 10.0 m^2^ are evaluated. The origin of the coordinate system is placed at the center of the xy‐plane, in a distance of 0.90 m above the floor. The height of the bunker is kept constant at 3.90 m for all bunker sizes.

Finally, histograms of the applied field sizes of each beam are created for the different collimator options of the CK‐M6. For the MLC fields, the diameter of a circle with the equivalent field size as the MLC opening is calculated.

Furthermore, the modulation factor (MF), as defined by Purwar et al^6^ is calculated for all treatments using the information stored in the treatment list:

(2) $$MF=\frac{Delivered\;MUs\;[MU]}{Prescribed\;Dose\;[cGy]}$$

## 3 RESULTS

**Figure 2:**
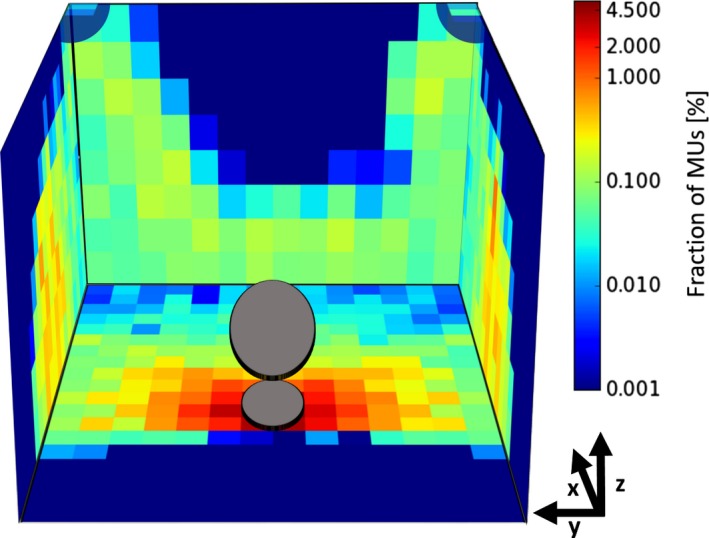
MU distribution for all beams. Overview of the MU distribution for all beams included in this study for our bunker. The semi‐transparent corners are the only locations that were hit by any beams. The wall behind the patient's head is not hit by any of the applied beams. In gray, the patient lying in head‐first‐supine position is indicated.

The resulting MU distribution for all primary radiation beams in the treatment list is visualized in Fig. 2 for the 9.5 × 7.0 m^2^ bunker. From this figure, it can be concluded that the applied MUs are neither distributed homogenously between the different barriers nor is the distribution homogenous on a single barrier itself. A more quantitative analysis is presented in Table 2, where the MU fractions for the four walls, the ceiling and the floor are listed. Furthermore, the results are separately shown for cranial as well as extracranial treatments sites. While the number of beams hitting the wall at the patient's feet and the ceiling drop to zero for extracranial treatments, about 13% more MUs are delivered to the floor, compared to the cranial treatments. Table 2 further shows MU distributions as differentiated by collimator system use.

**Table 2:** Filtered MU distributions for the wall, ceiling, and floor

Site	All	Cranial			Extracranial		
Collimator	All	All	Fix	Iris	All	Iris	MLC
Left	12.0%	12.3%	12.4%	12.1%	11.7%	11.5%	13.7%
Right	11.5%	12.7%	12.6%	12.7%	10.0%	9.6%	12.8%
Ceiling	<0.1%	0.1%	<0.1%	0.2%	0.0%	0.0%	0.0%
Floor	71.2%	65.4%	65.8%	64.9%	78.4%	78.9%	73.5%
Feet	5.3%	9.4%	9.1%	10.1%	0.0%	0.0%	0.0%
Head	0.0%	0.0%	0.0%	0.0%	0.0%	0.0%	0.0%

MU distribution for the different walls, ceiling, and floor. Furthermore, filters regarding the treatment site and collimator system were applied.

As there are only few irradiations using the Fix collimator for extracranial treatments and until now only few cases using the MLC for cranial treatments, the following situations are compared: Fix collimator vs. Iris collimator for cranial treatments and Iris collimator vs. MLC for extracranial treatments. The largest difference between collimators is, that using the MLC, about 5% more MUs are delivered to the left or right wall instead of to the floor.

**Figure 3:**
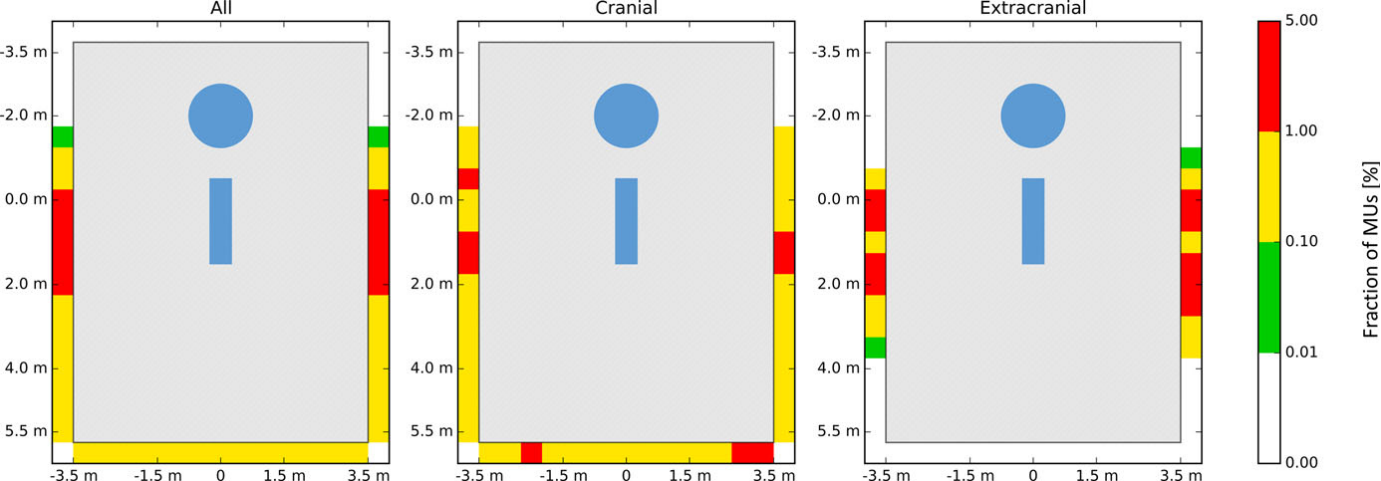
Bunker top‐view for different treatment sites. Top‐view of the bunker, showing the MU distribution for the different walls, integrated over their height. Left: Distribution for all beams. Middle: Cranial treatments. Right: Extracranial treatments. The blue circle and the blue rectangle indicate the position of the robot as well as the treatment couch inside the room.

The ‘top‐views’ in Fig. 3 show the overview of the MU distribution for all barriers simultaneously. In Fig. 3(a), the MU distribution including all cases is shown. In contrast to the results for the CK‐G4 by Yang and Feng,^6^ the distribution on the right wall and the left wall is more symmetric for the CK‐M6. Figures 3(b) and 3(c) show the distribution for cranial and extracranial irradiations in order to illustrate the differences due to the patient group. For both choices, an asymmetry is visible in the left wall vs. right wall. However, as shown in Table 2, the difference between the applied MUs to the left and the right wall is smaller for the cranial than for the extracranial irradiations: 0.3% vs. 1.7%. In order to determine whether the asymmetry in the extracranial distribution arises from the asymmetric position of the targets in the body, the delivered MUs for the left and right wall for different extracranial treatment sites are shown in Table 3. The differences in the total MU delivered to the left or right wall are more pronounced for the liver treatments than for lung, prostate, and spine treatments.

**Table 3:** MU distribution for different treatment sites

	Left	Right	Difference
Liver	12.0%	5.5%	6.5%
Lung	10.7%	8.8%	1.9%
Prostate	15.2%	12.5%	2.7%
Spine	11.0%	10.8%	0.2%

MU distribution for the left and right walls for different extracranial treatment sites.

The dependency of the MU distribution on the bunker size is presented in Table 4. Compared to a large bunker, a smaller bunker leads generally to larger MUs per pixel, as its inner surface area is smaller. Furthermore, the MU distribution on the wall at the patient's feet depends highly on the ratio of the bunker length from the origin on (x‐direction) and bunker width (y‐direction). As the bunker height is kept constant, the number of MUs hitting the ceiling is mainly dominated by the distance between the origin and the wall at the patient's feet.

**Table 4:** MU distribution for the different bunker sizes

	6.4 × 4.8 m^2^	6.4 × 6.4 m^2^	7.3 × 6.4 m^2^	9.5 × 7.0 m^2^	10.0 × 10.0 m^2^
Left	13.8%	10.9%	11.2%	12.0%	9.2%
Right	13.2%	10.3%	10.7%	11.5%	8.7%
Ceiling	0.0%	0.0%	0.0%	<1.0%	<1.0%
Floor	64.8%	68.9%	69.3%	71.2%	73.4%
Feet	8.3%	10.0%	8.9%	5.3%	8.6%
Head	0.0%	0.0%	0.0%	0.0%	0.0%

MU distribution for the different walls, ceiling, and floor for different bunker sizes. The names in the header line correspond to the bunker footprints. The expression ‘<1.0%’ means, that there are less than 1.0% but more the 0.0% of the MUs delivered.

**Figure 4:**
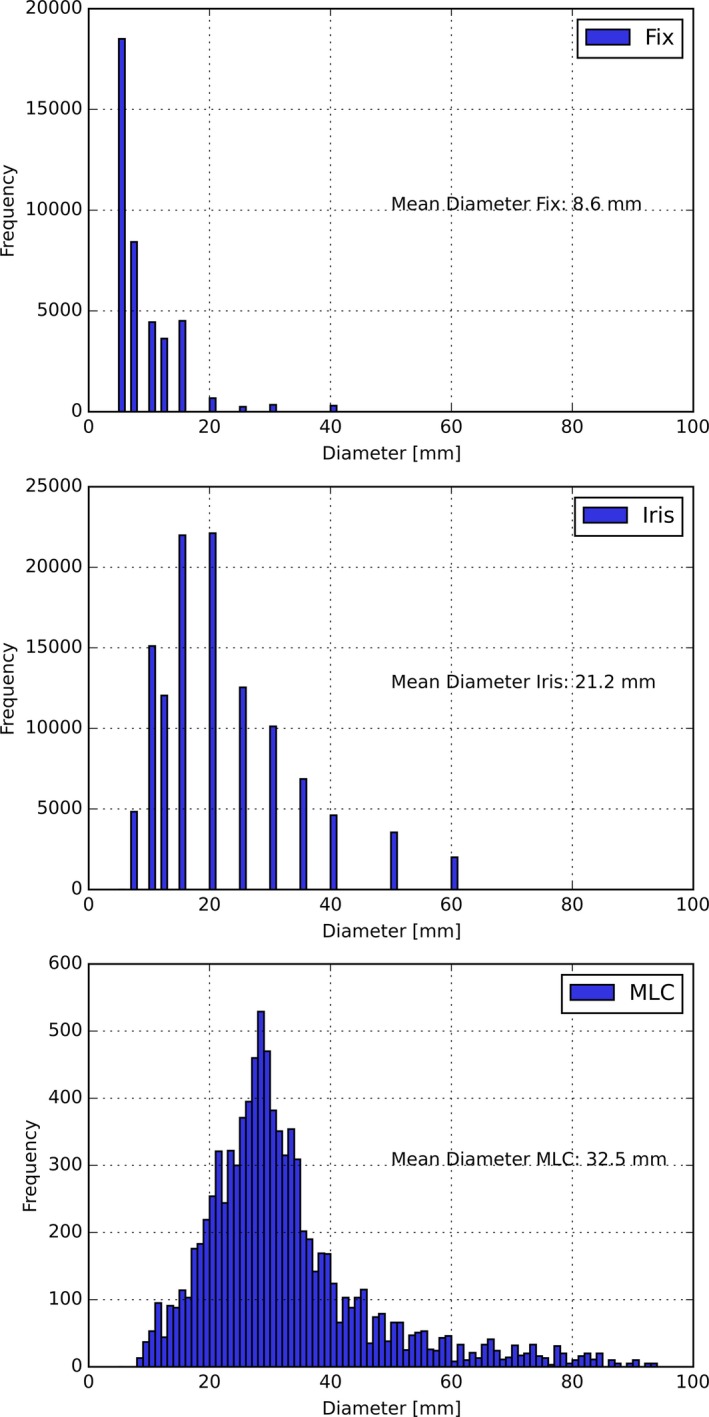
Applied field sizes for different collimators. Distribution of the diameters for the applied fields, filtered by the employed collimator system. For the MLC, the diameter for a circle with the equivalent field size as the MLC opening is shown.

The analysis of the applied field sizes, represented by histograms of the field diameters in Fig. 4, shows the continuous field sizes applied with the MLC versus the discrete field sizes for the two other collimation devices. As expected, Fix collimators are mainly used for the smallest available field sizes (5 and 7.5 mm diameter). Furthermore, there are MLC fields applied with openings that are beyond the largest possible Fix and Iris field size (Fig. 4).

**Figure 5:**
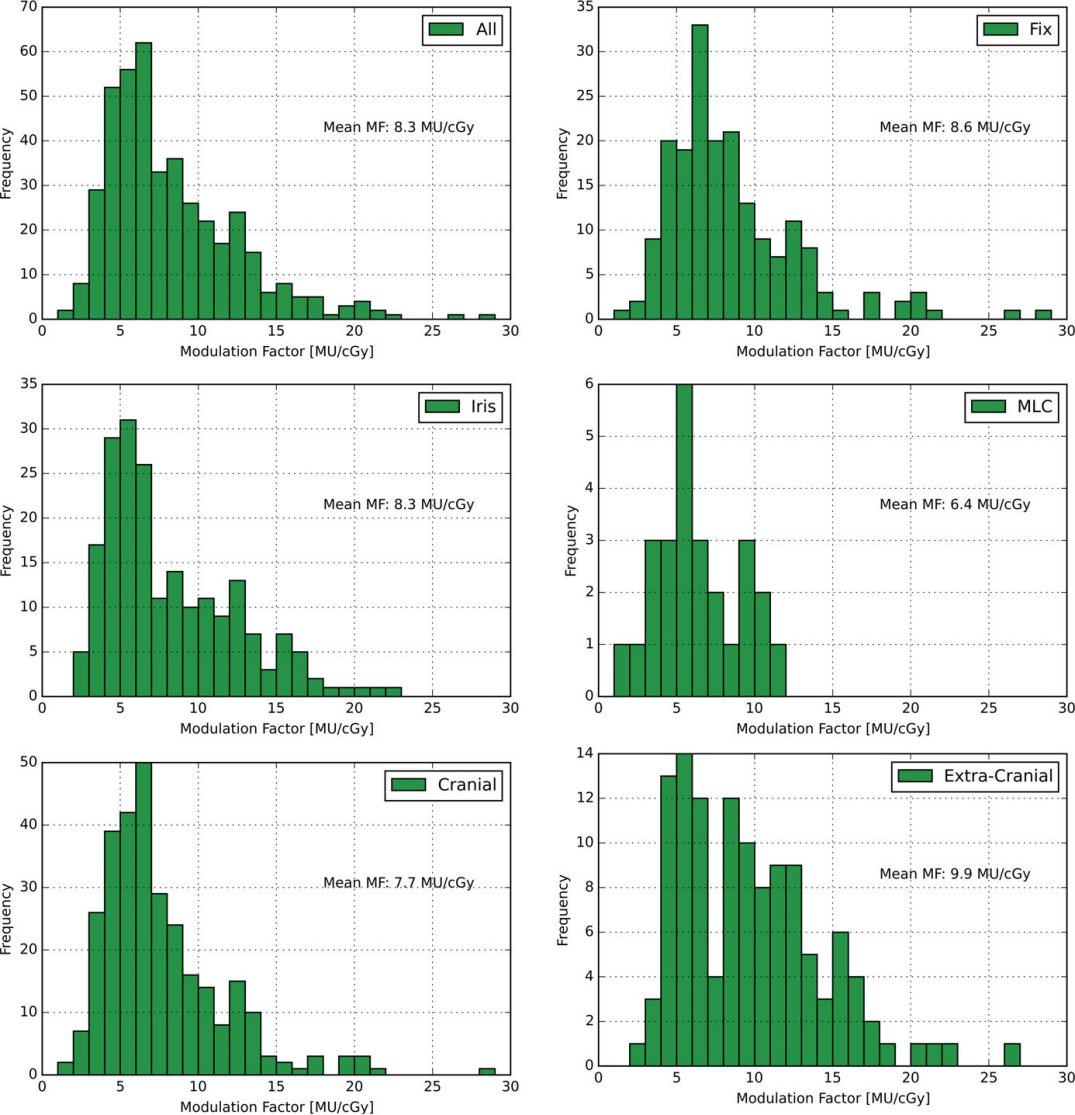
Modulation factors for different collimators and treatment sites. Distribution of the MF per used collimator system and treatment site. The MF is calculated by dividing the applied MUs per treatment plan by the prescribed dose [cGy].

Finally, MFs of the treatment plans for the different collimation devices and treatment sites are compared. While the mean MF for the Fix collimator is the highest, the MLC shows the smallest MF (Fig. 5). Although the extracranial treatments generally encompass larger field sizes, the mean MF is still higher than for cranial treatments (Fig. 5). Investigating the MF of the different extracranial treatment sites reveals that there are large differences between the mean MF for spine treatments (15.4 MU/cGy), prostate treatments (9.9 MU/cGy), liver treatments (7.2 MU/cGy), and lung treatments (7.5 MU/cGy).

## 4 DISCUSSION

A framework to evaluate important parameters regarding the radiation protection considerations of a CK‐M6 was developed. The resulting MU‐weighted direction distributions represent the current situation at our Institute and are very heterogeneous for the different barriers of the bunker. Whereas the left and right wall receive between 10% and 13% of the MUs irrespective of the collimator system or treatment site choice, the situation is different for the wall at the patient's feet. Cranial treatments result in a MU fraction of about 5% delivered to that wall, while extracranial treatments do not deliver direct beam there. This is explained analyzing the allowed beam directions for those two treatment types. As the CK‐M6 system just allows beams with an elevation up to 22° from the horizontal direction, the low number of MUs for ceiling is expected. However, for cranial treatments some beams hit the corners of the ceiling. The floor and the wall behind the patient's head represent the extreme values in this study, receiving 65%–79% and 0% of the MUs, respectively. Even if the orientation of the CK‐M6 and the location of the adjacent rooms are already fixed, the varying MU distribution over the barriers allows identifying suitable places to create e.g., a cable duct.

The enhanced symmetry in the node distribution for the CK‐M6 compared to the CK‐G4 explains the observed differences in the right and left wall MU distributions between the work of Yang and Feng^6^ and this study. The anatomical location of target volumes within the patient may further explain the differences in right and left wall MU distributions in extra‐cranial treatments.

Generally, the different collimator systems used had a minor effect on the direction distribution, compared to the influence of different treatment sites.

While enlarging the area that has to be shielded, larger bunkers naturally lead to a smaller number of MU/pixel. Furthermore, bunkers with a nonsquared footprint lead to substantial changes in the MU ratio between the left/right wall vs. the wall at the patient's feet and floor.

Regarding the secondary radiation, the mean MF for the Fix and Iris collimators (8.6 and 8.3 MU/cGy) are larger than the mean MF for the MLC of 6.4 MU/cGy. This is due to the segmented manner of delivery as well as the larger field sizes, which are offered by the MLC and also used during the treatments (Fig. 5). In addition to the collimator system choice, the treatment site has a major influence on the MF.

So far, all clinically delivered beams at our institution are included in the database. By always incorporating the most recent treatments, it is possible to monitor the radiation protection issues in almost real‐time. Furthermore, the whole framework was developed in a two‐step approach, in which the creation of the treatment list removes any sensitive information. This allows easily comparing data for different centers in future works.

## 5 CONCLUSIONS

The developed framework allows analyzing and monitoring radiation protection parameters for the present situation as well as filtering for collimators or treatment sites and exploring different bunker sizes.

## CONFLICT OF INTEREST

The authors declare that they have no conflicts of interest.
